# Hayai-Annotation: A functional gene prediction tool that integrates orthologs and gene ontology for network analysis in plant species

**DOI:** 10.1016/j.csbj.2024.12.011

**Published:** 2024-12-16

**Authors:** Andrea Ghelfi, Sachiko Isobe

**Affiliations:** a Bioinformation and DDBJ Center, National Institute of Genetics, Yata, 1111, Mishima, Shizuoka 411-8540, Japan; b Kazusa DNA Research Institute, Kazusa-Kamatari, 2-6-7, Kisarazu, Chiba 292-0818, Japan; c Graduate School of Agricultural and Life Sciences, The University of Tokyo, 1-1-1, Yayoi, Bunkyo, Tokyo 113-8657, Japan.

**Keywords:** Functional annotation, Network analysis, GO enrichment, Ortholog inferences, Co-occurrence of orthologs and Gene Ontologies

## Abstract

Hayai-Annotation, an annotation tool powered by the R-shinydashboard browser interface, implements a workflow that integrates sequence alignment using DIAMOND against UniProtKB Plants and ortholog inference using OrthoLoger. We here propose a pipeline to explore genome evolution and adaptation from a different perspective, by creating a network considering orthologs and gene ontology as nodes, with edges based on the annotation for each gene. This approach aims to improve the visualization of conserved biological processes and functions, highlight species-specific adaptations, and enhance the ability to infer the functions of uncharacterized genes by comparing edge patterns across species. To our knowledge, this is the first attempt to build a network using annotated OrthoDB orthologs and Gene Ontology terms (Molecular Function and Biological Process) as nodes, providing a comprehensive view of gene distribution and function in plant species. The GO annotation accuracy was assessed by the CAFA-evaluator, demonstrating that the accuracy of this version of Hayai-Annotation exceeded that of the benchmark, InterProScan. The updated Hayai-Annotation enhances ortholog analysis functionality, allowing for evolutionary insights from gene sequences, and is expected to contribute significantly to the future development of plant genome analysis.

## Graphical Abstract

**Figure:**
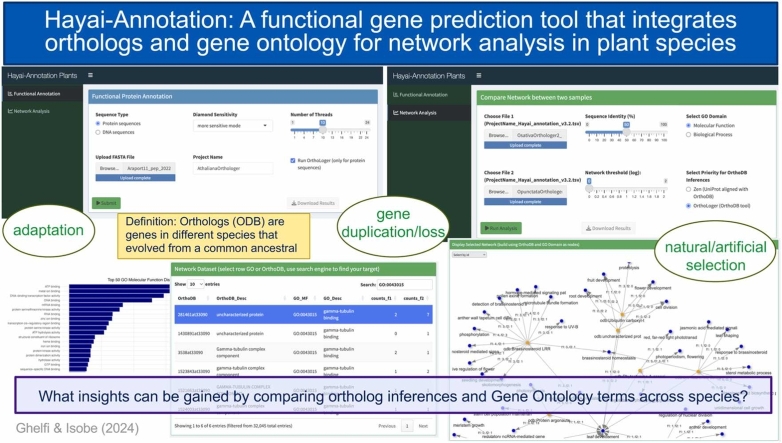

## 1 Introduction

The advent of next-generation sequencing (NGS) and advances in bioinformatics for genome sequence analysis have led to an exponential growth in plant genome information [1]. This surge in genomic data is crucial for accelerating progress in plant research, as it enables molecular biologists to obtain extensive and accurate knowledge of gene profiles in relevant genomes. Numerous tools are available for the annotation of gene and protein functions, such as OmicsBox/Blast2GO [2], InterProScan [3], Mercator [4], and FunctionAnnotator [5]. However, these tools do not extensively leverage evolutionary relationships that are specifically tailored for ortholog detection.

Meanwhile, with the increasing availability of diverse plant genome sequences, cross-species gene conservation analysis has become more prevalent. Moreover, as described by Gabaldon and Koonin [6], orthology is the most accurate way to describe differences and similarities in the composition of genomes from different species. Orthologs trace back to an ancestral gene that was present in a common ancestor of the compared species, and thus they tend to retain ancestral functions [6].

The various software tools available for ortholog detection include OrthoLoger [7], OrthoFinder [8], OrthoMCL [9], and EggNOG-mapper [10]. OrthoDB, a leading resource for precomputed gene orthology, implements OrthoLoger, which is considered a state-of-the-art software for orthology inference [11]. While OrthoDB aspires to encompass all species in order to facilitate accurate comparative research, the expanding data volume presents computational challenges [11].

However, inferring orthologous genes is challenging, particularly in cases involving orphan genes (genes without detectable homologs in other species), differential gene loss [12], horizontal gene transfer [13], and domain shuffling [14]. These evolutionary events can obscure orthologous relationships and complicate the accurate identification of true orthologs. Moreover, sequence alignment methods have inherent limitations, especially when dealing with distant homologs or complex gene evolution scenarios. Therefore, integrating multiple analytical approaches provides a more robust framework for understanding the complexity of biological systems.

Here, we present Hayai-Annotation v3, an updated [15] standalone application with a browser-based interface that was developed using R-shinydashboard [16] and specifically designed to address these challenges in plant genome annotation. This version introduces several key innovations and is now composed of two modules. The first of these is the Functional Annotation module, which (1) incorporates ortholog information by integrating OrthoLoger [7] to map orthologs to OrthoDB v12 [11]; (2) implements DIAMOND [17] to align sequences against the UniProtKB-Plants [18](Viridiplantae) database, which now includes algae in recognition of the evolutionary importance of algae as ancestral photosynthetic organisms and their relevance in comparative genomics; (3) introduces a pre-computed mapping dataset built by aligning UniProt-Plants with OrthoDB-Plants, named Zen, addressing the computational challenges associated with traditional ortholog detection tools and providing an alternative method for ortholog inferences; and (4) introduces the second of the modules in Hayai-Annotation v3, the Network Analysis module, which offers an interface for comparative annotation analysis between two species.

The Network Analysis module aims to: (a) compare OrthoDB IDs assigned by Zen and OrthoLoger between species to help infer descriptions for uncharacterized sequences or ortholog groups (OGs); (b) compare Gene Ontology (GO) terms associated with OGs in two samples to discuss potential gene function propagation; and (c) analyze OGs between two species to explore potential gene loss or gain events.

To our knowledge, Hayai-Annotation v3 represents the first attempt to construct networks using OrthoDB descriptions and GO terms as nodes, particularly when employing an independent method for their respective inferences. The Network Analysis module facilitates visualization of conserved biological processes and species–specific adaptations, providing a comprehensive view of gene distribution and function in plant genomes. By integrating robust annotation methods with innovative comparative analysis capabilities, Hayai-Annotation v3 addresses critical gaps in plant genome annotation and offers valuable tools for the plant genomics community.

## 2 Materials and methods

### 2.1 Dataset sources and processing

The UniProtKB-Plants [18] dataset, consisting of uniprot_sprot_plants.dat.gz and uniprot_trembl_plants.dat.gz, was downloaded from https://ftp.uniprot.org/pub/databases/uniprot/current_release/knowledgebase/taxonomic_divisions/. The OrthoDB v12 dataset was downloaded from https://data.orthodb.org/v12/download/. Taxonomic information was downloaded from ftp://ftp.ncbi.nih.gov/pub/taxonomy/taxdump.tar.gz to identify plant species within the OrthoDB v12 dataset. All data was downloaded on October 13, 2024, and the downloaded files underwent MD5sum verification.

The information from UniProtKB, such as sequences in FASTA format, accessions, ODB ID, Gene Ontology (GO), Pfam, InterPro and Protein Existence (PE) IDs, was extracted from UniProt “dat” files using *in-house* BioPython scripts.

### 2.2 Peptide sequence datasets

The performance of Hayai-Annotation was evaluated using the peptide sequences of *Arabidopsis thaliana*, *Oryza sativa*, and *Oryza punctata* as described below. All files were downloaded on October 20, 2024.

*A. thaliana*:

https://www.arabidopsis.org/download/file?path=Proteins/Araport11_protein_lists/Araport11_pep_20220914_representative_gene_model.gz

Domesticated rice, *O. sativa* Japonica Group, Os-Nipponbare-Reference-IRGSP-1.0:

https://rapdb.dna.affrc.go.jp/download/archive/irgsp1/IRGSP-1.0_protein_2024-01–11.fasta.gz

Wild-type rice, *O. punctata*:

https://ftp.ensemblgenomes.ebi.ac.uk/pub/plants/release-59/fasta/oryza_punctata/pep/Oryza_punctata.Oryza_punctata_v1.2.pep.all.fa.gz

### 2.3 Construction of the Zen mapping dataset and integration into the annotation pipeline

To provide an alternative method for ortholog inference in the annotation process, we constructed a precomputed mapping dataset called Zen. Zen was developed by aligning UniProtKB-Plants sequences with OrthoDB-Plants using USEARCH [19]. Initially, we extracted plant species sequences from the OrthoDB repository to create OrthoDB-Plants, which served as the primary reference database. We then aligned sequences from UniProtKB-Plants against OrthoDB-Plants utilizing USEARCH v11 (64-bit version), with the following alignment parameters: sequence identity of 50 % or more, E-value lower than 10^−6^ and coverage equal or exceeding 75 % for both target and query sequences. In this process, the OrthoDB ID from the best hit in OrthoDB-Plants was propagated to the corresponding UniProt accession. We refer to the OrthoDB ID assigned through this method as Zen_OrthoDB to distinguish it from ODB_OG, which is obtained via OrthoLoger, and from the orthologs group (OG) annotations directly available from UniProtKB, which are referred to as UKB_OrthoDB.

The resulting mapping between UniProt accessions and OrthoDB IDs (Zen_OrthoDB) was integrated into the headers of the FASTA file used as the reference database in Hayai-Annotation v3, together with the annotation layers extracted directly from UniProtKB, such as GO, InterPro, Pfam, Evidence existence and OrthoDB. This integration allows the annotation pipeline to rapidly assign all the annotation layers during sequence alignment, improving its efficiency.

### 2.4 Hayai-Annotation modules

Hayai-Annotation v3 has a browser-based interface developed using the R package shinydashboard (v0.7.2) [16]. It has two modules, the Functional Annotation module and the Network Analysis module. Available on GitHub as a Conda [20] environment, it includes JupyterLab [21] for easy setup and seamless access on remote servers (https://github.com/aghelfi/HayaiAnnotation).

#### 2.4.1 Functional Annotation module

The Functional Annotation module utilizes a workflow that integrates DIAMOND [22] (v2.1.9) for sequence alignment against UniProtKB-Plants as a reference database and employs the orthomapper function from OrthoLoger [7] (v3.5.0, https://orthologer.ezlab.org/) using the node Viridiplantae to detect orthologs.

The Hayai-Annotation v3 browser implements the option for all seven sensitivity options in DIAMOND, ranging from "fast" mode (less accurate) to "ultra-sensitive" mode (more accurate). For sequence alignment, the module accepts DNA sequences and performs translated searches (similar to BLASTX). If the input consists of protein sequences, users have the option to include OrthoLoger inferences by clicking the "Run OrthoLoger" check box. Users can set the number of threads and the project name, then click "Submit" to start the analysis. Upon completion, the main output table is displayed with most of its columns in the Annotation Output section. To download all the files, click on "Download Results"; the downloaded ZIP file will follow the pattern ProjectName_HayaiAnnotation_v3.2.zip.

The output file in zip format contains:

• Main annotation file: “ProjectName_Hayai_annotation_v3.2.tsv”.
• GO and InterPro counts: Four tables with counts of IDs and terms for the three GO domains (MF, BP, and CC) and InterPro, all in TSV format.
• Top 50 term graphics: Four graphics showing the top 50 terms for GO (MF, BP, and CC) and InterPro in PDF format.
• Unaligned sequences: A FASTA file unaligned.fasta containing sequences not aligned by DIAMOND.
• Log files: Logs documenting the analysis process.

The main file “ProjectName_Hayai_annotation_v3.2.tsv” contains different columns depending on whether OrthoLoger is used. When annotated without OrthoLoger, the columns are Query, Accession, Product_Name, OrthoDB, OrthoDB_Desc, UKB_OrthoDB, GO_BP, GO_MF, GO_CC, Evidence_existence, InterPro, Pfam, Identity, Length, Evalue, and Score. In this case, the OrthoDB entries are provided solely by Zen’s mappings. The original OG ID from UniProtKB, when available, is listed under UKB_OrthoDB. When OrthoLoger is included in the annotation, the columns are expanded to include Zen_OrthoDB, Zen_OrthoDB_Desc, ODB_OG, ODB_Description, ODB_GO_MF, ODB_GO_BP, ODB_EC, ODB_COG_category, ODB_KEGG_ko, ODB_Interpro, ODB_evalue, and ODB_score, plus all columns from the non-OrthoLoger annotation. In this scenario, OrthoDB is set to prioritize OGs inferred from OrthoLoger. When Gene Ontology terms for Biological Process (BP) or Molecular Function (MF) are absent in UniProtKB accessions, the corresponding GO_BP or GO_MF values are propagated from OrthoLoger inferences (ODB_GO_MF and ODB_GO_BP).

#### 2.4.2 Network analysis module

The Network Analysis module allows comparison between two species annotated using Hayai-Annotation v3. It uses the file “ProjectName_Hayai_annotation_v3.2.tsv” as input. Users can select the values for the following parameters:

• Sequence Identity: Filters sequences based on the “Identity” parameter from the DIAMOND alignment.
• GO Domain: Offers two options—Molecular Function (MF) or Biological Process (BP).
• Priority for OrthoDB Inferences: Determines which source has priority in assigning OrthoDB IDs, using the OrthoLoger tool (ODB_OG) or the Zen pre-computed mappings (Zen_OrthoDB).
• Network threshold (log scale): Sets the difference in logarithmic scale (ln + 1) for the counts of all co-occurrences between GO terms and OrthoDB values for each species.

It is important to note that even if OrthoLoger was selected as the priority in the main annotation file, this module allows users to change the priority setting. The only values that change based on this priority are those in the OrthoDB and OrthoDB_Desc columns, which are updated according to the priority set in this module.

This module comprises five parts, which are known as: (1) input files (f1 and f2) and parameters selections; (2) the Network Dataset; (3) the Network Visualization; and (4) and (5) the tables from the original uploaded files in this section, respectively.

The Network Dataset is a table generated based on user parameter selections. It displays all the co-occurrences of OrthoDB orthologous groups (OGs) and Gene Ontology (GO) terms, either Molecular Function (MF) or Biological Process (BP), along with the counts for each species. It is important to note that although the Network threshold is set on a logarithmic scale as a normalization method to compare both species, the values in the Network Dataset represent the absolute counts for each species. Users can utilize the search engine to look for orthologs descriptions, Gene Ontology terms, or their associated IDs. Downloading is available for the options selected using the “Download Results” button.

The module is directed to interactively build a network by clicking on a row in the Network Dataset table. It uses the GO term from the selected row to find all the corresponding orthologs, then connects these orthologs with all the Gene Ontology terms associated with them. In this approach, both GO terms and OGs are considered nodes in the network, and the edges correspond to the counts of genes for each OG–GO pair, formatted as f1:counts_f1, f2:counts_f2.

The network is constructed with 100 nodes or less to maintain clarity. To facilitate identification within the network, GO descriptions are truncated to 30 characters, and OG descriptions to 20 characters. Since some OGs have the same or similar descriptions, truncated values might be identical; as a result, the network may display more than one edge per OG–GO pair. This feature can be insightful particularly when comparing the relationships between OGs and GO terms within different orthologs with similar descriptions.

In the network visualization, orthologs are represented by orange dots with the prefix "odb" (referring to OrthoDB) before the ortholog description, while GO terms are represented by blue dots. Arrows indicate the direction from orthologs to Gene Ontology terms. The network graph was constructed using the R package visNetwork (2.1.2) [23].

### 2.5 Evaluation and benchmarking of GO predictions

To assess the performance of Hayai-Annotation v3 in predicting Gene Ontology (GO) terms, we employed the CAFA-evaluator tool (available at https://github.com/BioComputingUP/CAFA-evaluator) [24]. This tool calculates weighted precision (proportion of correct positive predictions out of all positive predictions), recall (proportion of positive predictions made out of all positive examples in the dataset), and F-score (harmonic mean of precision and recall) independently for each of the three GO domains: Molecular Function (MF), Biological Process (BP), and Cellular Component (CC).

As a benchmark, we used InterProScan (v5.70; https://ftp.ebi.ac.uk/pub/software/unix/iprscan/5/5.70–102.0/interproscan-5.70–102.0-64-bit.tar.gz) [3], a widely recognized platform for functional annotation and GO mapping. To avoid sample biases and ensure statistical robustness, we extracted three independent samples of 1000 genes each from *A. thaliana*. The GO annotations provided by the Gene Ontology Consortium [25] for *A. thaliana* (https://current.geneontology.org/annotations/tair.gaf.gz) are considered the gold standard for evaluating prediction accuracy; they were downloaded on October, 20, 2024.

### 2.6 Evaluation of Zen OrthoDB mapping

#### 2.6.1 Benchmarking UniProtKB

To assess the accuracy of the Zen_OrthoDB mappings, we conducted a comparative analysis between the orthologous groups (OGs) inferred by Zen and the original OGs annotated in UniProtKB. Given that Zen has recently been updated to OrthoDB v12, while UniProtKB OrthoDB annotations remain based on OrthoDB v11, we performed the evaluation using Zen OrthoDB mappings using our previous version of Zen, which was based on OrthoDB v11.

We began by extracting a total of 2729,135 UniProtKB-Plants accessions that were originally annotated with OrthoDB IDs. Using the "odb11v0_OG_pairs.tab" file provided by OrthoDB v11, we identified accessions with corresponding parental OGs at the taxonomic levels of Viridiplantae (taxid 33090) and Eukaryota (taxid 2759). This process resulted in the identification of 1950,290 accessions, representing 71.5 % of the total, that had such correspondences.

Focusing on these 1950,290 accessions with corresponding parental OGs, we compared the OrthoDB IDs assigned by Zen_OrthoDB with the original OrthoDB IDs annotated in UniProtKB for each accession. We calculated the percentage of exact matches between the Zen_OrthoDB inferred IDs and the original annotations to evaluate the concordance between the two sets of annotations. The scripts used for this analysis are described in the Supplementary File S1.

#### 2.6.2 Semantic similarity score

To assess the semantic similarity between annotated protein product names by UniProtKB and their corresponding OrthoDB descriptions inferred using Zen mapping, we employed BioBERT [26], a pre-trained biomedical language model optimized for natural language processing tasks in the biomedical domain. Specifically, we used the output file from Hayai-Annotation for *Arabidopsis thaliana*, focusing on the “Product_Name” and “Zen_OrthoDB_Desc” columns. We filtered out entries labeled as “uncharacterized” or “hypothetical” proteins and those lacking clear descriptions, such as proteins identified only by “Emb_” or “Gb_” prefixes. Utilizing the SentenceTransformer model “pritamdeka/BioBERT-mnli-snli-scinli-scitail-mednli-stsb”, we computed similarity scores between the remaining pairs of product names and OrthoDB descriptions. A similarity threshold of 0.5 was established to determine significant semantic alignment. The distribution of similarity scores was visualized using a histogram plot generated with matplotlib [27].

### 2.7 EggNOG-mapper annotation

EggNOG-mapper version 2.1.12 (https://github.com/eggnogdb/eggnog-mapper) [10] was used to annotate peptide sequences from *A. thaliana*, *O. sativa* and *O. punctata*. The software was installed via pip and executed using its default parameters to ensure consistency with its standard annotation workflow.

## 3 Results and discussion

### 3.1 Expanding OrthoDB coverage in UniProtKB-Plants using Zen mapping

The UniProtKB-Plants database assigns an OrthoDB orthologous group (OG) to only 14.3 % of accessions, comprising 50.8 % of UniProt Swiss-Prot accessions (22,658 accessions) and 14.2 % of UniProt TrEMBL accessions (2706,477 accessions). To expand the coverage of plant-specific orthologs in UniProtKB-Plants, we aligned UniProtKB-Plants with OrthoDB-Plants. After alignment, there was a significant increase in UniProt accessions assigned an OrthoDB ID, from 14.3 % to 48.3 % (Table 1), and we called this correspondence Zen mapping.

**Table 1:** Comparison of OrthoDB Orthologous Group Assignments in UniProtKB-Plants Before and After Zen Mapping*.

Database source (UniProtKB-Plants)	Number of accessions with OrthoDB ID - UniProtKB (%)	Total number of accessions with OrthoDB ID - Zen Mapping (%)	Total Number of accessions
Swiss-Prot	22,658 (50.8)	38,765 (86.9)	44,592
TrEMBL	2706,477 (14.2)	9170,680 (48.2)	19,023,625
Total	2729,135 (14.3)	9209,445 (48.3)	19,068,217

* UniProtKB utilizes information from OrthoDB v11 for assigning OrthoDB IDs. To ensure accurate comparisons, the Zen Mapping values were also generated using OrthoDB v11.

### 3.2 Evaluation of Zen mapping

#### 3.2.1 Benchmarking UniProtKB

The comparison between the OGs inferred using Zen, called Zen_OrthoDB, and the original OGs annotated in UniProtKB revealed a high level of concordance. Out of the 1950,290 accessions with corresponding parental OGs, 1943,023 accessions (99.6 %) had exactly the same OGs assigned by both Zen_OrthoDB and UniProtKB. This high degree of agreement demonstrates that the Zen mapping reliably replicates existing OGs annotations.

#### 3.2.2 Semantic similarity score

**Fig. 1:**
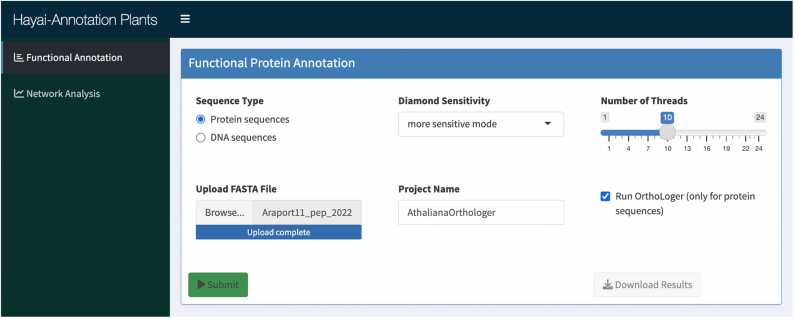
Interface of the functional annotation module in Hayai-Annotation.

From the analysis of 20,490 pairs of product names in the UniProtKB and OrthoDB product descriptions from the annotation of *A. thaliana*, we found that 15,617 pairs (76.22 %) exhibited similarity scores above the threshold of 0.5, indicating significant semantic similarity. The mean similarity score was 0.7104, and the median was 0.7777, reflecting a strong overall alignment between the product names and the Zen-inferred descriptions (Supplementary File S1 Fig. 1).

These findings highlight the effectiveness of Zen mapping in providing meaningful ortholog descriptions that closely align with existing protein annotations. The high proportion of similar pairs demonstrates that Zen mapping can reliably infer orthologous relationships with semantic consistency to known protein functions. Although identical nomenclature between product names and ortholog descriptions is not necessarily expected, this method provides strong support for meaningful alignment. The use of BioBERT for similarity assessment allowed for nuanced evaluations, capturing semantic relationships beyond exact string matches and accounting for synonyms and related terms prevalent in biological nomenclature.

### 3.3 Gene ontology prediction evaluation

The evaluation of GO predictions was conducted using the CAFA-evaluator on annotation results from three replicates of 1000*A. thaliana* genes. For this evaluation, Hayai-Annotation was executed with DIAMOND in “more sensitive” mode, without OrthoLoger, and its performance was compared to that of InterProScan. Annotations from the Gene Ontology were used as the benchmarking gold standard. Weighted precision, recall, and F-scores were calculated independently for each GO domain to provide an objective comparison of tool performance across the Biological Process, Molecular Function, and Cellular Component domains.

The results demonstrated that Hayai-Annotation achieved higher mean weighted F-scores across all GO domains compared to InterProScan (Table 2). This advantage was likely due to the high sensitivity of DIAMOND and the robust GO annotation quality in UniProtKB. It is important to note that, despite achieving lower scores, InterProScan remains a valuable resource for gene annotation due to its extensive database integration of multiple databases across diverse taxonomic domains. Additionally, the recent update to tair.gaf by geneontology.org (formerly goa_arabidopsis.gaf) may have introduced more manually curated annotations, potentially increasing the challenge for software tools to accurately predict these annotations.

**Table 2:** Mean weighted F-scores ( ± Standard deviation) for each tool and GO domain.

Tool	Gene Ontology Domain		
	BP	MF	CC
Hayai-Annotation	0.7054 (0.0111)	0.6233 (0.0047)	0.5703 (0.0153)
InterProScan	0.4749 (0.0107)	0.5468 (0.0114)	0.2935 (0.0043)

### 3.4 Hayai-Annotation: Functional annotation module

The new interface of Hayai-Annotation, shown in Fig. 1, includes options to run in all available sensitivity settings in DIAMOND (v2.1.9) and incorporates an option to run OrthoLoger (v3.5) at the Viridiplantae level. To demonstrate the functionalities of Hayai-Annotation, in addition to the complementary roles of Zen mappings and OrthoLoger, we annotated three plant species—*A. thaliana*, *O. sativa*, and *O. punctata*—using DIAMOND’s “more sensitive” mode and enabling the “Run OrthoLoger” option.

The proportion of genes assigned to OGs by both methods, by either method exclusively, or those left unassigned by both methods were compared (Table 3). This analysis provides insights into the potential of Zen mapping and OrthoLoger to cover different gene sets, highlighting how each tool may contribute uniquely to comprehensive OG assignment across distinct plant genomes.

**Table 3:** Comparison of orthologous group (OG) assignments by zen mappings and orthologer across three plant species based on functional annotations from Hayai-Annotation v3.

Species	Genes assigned OGs by both methods (%)	Genes assigned an OG only by Zen (%)	Genes assigned an OG only by OrthoLoger (%)	Genes with no OG assigned by either method (%)^*1^	Unaligned genes (%)^*2^	Total number of genes
*A. thaliana*	84.8	10.9	1.1	3.2	0.2	27,562
*O. sativa*	60.7	11.4	4.6	23.3	0.1	42,665
*O. punctata*	58.4	9.8	10.7	21.1	0.2	41,060

^*1^ Genes without an OG assignment but annotated with UniProt accessions and associated annotations, including product names, GO terms, Pfam entries, and InterPro entries.

^*2^ Genes not aligned by DIAMOND and listed on unaligned FASTA file.

In *A. thaliana*, a well-studied model organism with extensive functional characterization and database support, both Zen mapping and OrthoLoger effectively captured OGs for a significant proportion of genes, covering 84.8 % of the total. Zen mapping provided unique annotations for an additional 10.9 % of genes, while OrthoLoger uniquely annotated 1.1 %. Only 3.2 % of genes remained unannotated by either method, demonstrating the overall comprehensive coverage achieved for this species.

For *O. sativ*a and *O. punctata*, the proportions of genes with OG assigned by both methods were lower, 60.7 % and 58.4 %, respectively. In *O. sativa*, Zen mappings uniquely annotated 11.4 % of genes, surpassing the unique annotations by OrthoLoger (4.6 %). Conversely, in *O. punctata*, OrthoLoger uniquely annotated a larger percentage of genes (10.7 %) compared to Zen mapping (9.8 %).

Zen mapping’s higher contribution of uniquely annotated genes, particularly in *A. thaliana* and *O. sativa*, underscores its advantage in providing complementary OG annotations for model organisms. This suggests that Zen mapping may leverage more studied species, enhancing its ability to annotate genes beyond the scope of OrthoLoger. In contrast, for *O. punctata*, OrthoLoger's higher contribution of uniquely annotated genes (10.7 % of genes) highlights its ability to capture genes that are potentially more taxonomically divergent.

These findings demonstrate that Zen mapping and OrthoLoger complement each other in annotating orthologs in plant genomes. The higher proportions of genes uniquely annotated by one method, particularly in *O. punctata* but also in *O. sativa*, suggest that these species benefit from the integration of multiple ortholog inference approaches. Employing both methods enhances the overall annotation coverage, especially for species with relatively less database representation.

The comparatively lower percentages of assigned orthologous groups in the *Oryza* species (23.3 % for *O. sativa* and 21.1 % for *O. punctata*) relative to *A. thaliana* may reflect a less comprehensive representation in current databases or indicate a higher level of genomic complexity. Still, it is noteworthy that fewer than 0.3 % of the genes remained completely unannotated (Table 3). For genes without OG assignments, the corresponding UniProt accessions and associated annotations (including product names, GO terms, Pfam, and InterPro entries) were still provided, ensuring that functional insights remain accessible despite the absence of OG information. See the project’s GitHub repository (https://github.com/aghelfi/HayaiAnnotation) for unaligned FASTA files and for genes annotated without OG, which are located in the “annotations” and “evaluation” directories, respectively.

We compared the performance of Hayai-Annotation and EggNOG-mapper by analyzing their OG assignments across three plant species (Table 4). This comparison includes the proportion of genes assigned to OGs by both tools, unique assignments made by each, and the number of unassigned genes. EggNOG-mapper showed broader coverage of orthologous groups, resulting in more annotated genes by including sequences with lower identities or distant homologs. Data examples generated using the UniProt “Align” tool (https://www.uniprot.org/align/) are available in the “analysis” directory of our GitHub repository. In contrast, Hayai-Annotation’s higher specificity in its orthology assignments is particularly valuable for comparative studies between species, as demonstrated by the Network Analysis module of Hayai-Annotation, which facilitates the exploration of uncharacterized genes while providing insights into conserved biological processes and species-specific adaptations through ortholog comparisons.

**Table 4:** Comparison of orthologous group (OG) assignments by Hayai-Annotation and EggNOG-mapper across three plant species.

Species	Genes assigned OGs by both tools (%)	Genes assigned an OG only by Hayai-Annotation (%)	Genes assigned an OG only by EggNOG-mapper (%)	Genes with no OG assigned by either method (%)	Total number of genes
*A. thaliana*	92.7	3.9	0.9	2.5	27,562
*O. sativa*	75.9	0.7	9.5	13.9	42,665
*O. punctata*	78.2	0.6	11.0	10.2	41,060

It is important to note that orthologous group inferences are approximations of complex evolutionary relationships and are not meant to provide unique solutions. Discrepancies between computational methods are expected due to differences in algorithms, reference databases, and criteria for orthology assignment [28]. These discrepancies are not mere inconsistencies but reflect the multifaceted nature of genome evolution, including gene duplication, loss, and lineage-specific expansions. The integration of multiple ortholog inference methods can therefore provide a more nuanced and comprehensive characterization of genes.

### 3.5 Hayai-Annotation: network analysis module

**Fig. 2:**
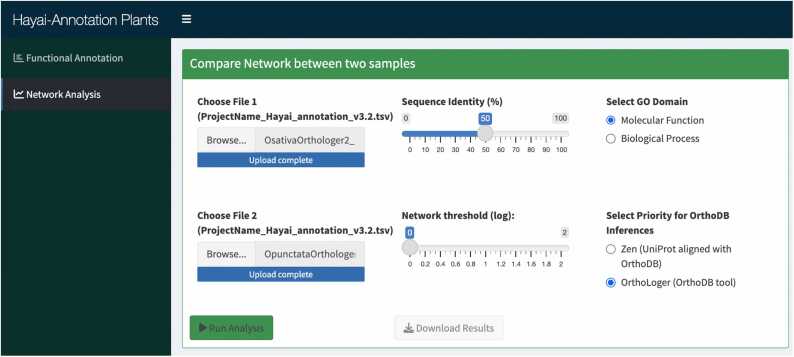
Interface of the network analysis module in Hayai-Annotation, displaying the uploaded files of *O. sativa* and *O. punctata* and the parameters sequence identity of 50 %, GO molecular function and OrthoLoger priority for OG inferences.

The Network Analysis module, a new feature in this version of Hayai-Annotation, enables comparisons of GO–OG term relationships, annotated for each protein, between two species annotated by Hayai-Annotation v3. This module takes the main annotation file generated at the Functional Annotation module as input (Fig. 2). To showcase the capabilities of the network analysis module, we examined the annotation files of *O. sativa* and *O. punctata* using the “more sensitive” mode of DIAMOND and including OrthoLoger.

We demonstrated three potential applications of this module: (1) enhancing the characterization of previously uncharacterized protein or orthologs groups; (2) comparing GO annotations across ortholog proteins between two species; and (3) exploring potential instances of gene loss or gain.

#### 3.5.1 Characterization of uncharacterized proteins

**Fig. 3:**
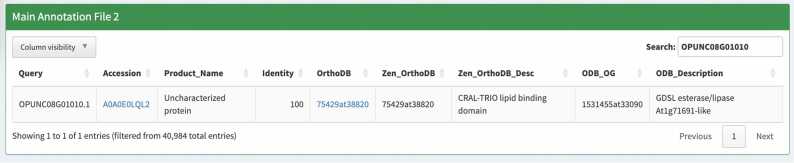
Main annotation File 2 is available at the bottom of the network analysis module, enabling easy searches for proteins and descriptions. The dataset is filtered based on the selections made at the top of the webpage, where OrthoLoger is set as the priority for OG assignment.

We take the characterization of the uncharacterized protein OPUNC08G01010.1 (UniProt accession A0A0E0LQL2) in *O. punctata* as an example. Hayai-Annotation inferred the OGs “GDSL esterase/lipase At1g71691” (1531455at33090) by OrthoLoger and “CRAL-TRIO lipid binding domain” (75429at38820, Poales level) by Zen (Fig. 3). Clicking the hyperlink in the OrthoDB column in the Main Annotation File 2, which corresponds to the *O. punctata* annotation file used as input in this analysis, causes a browser to open the OrthoDB v12 webpage (https://www.orthodb.org/?query=75429at38820), showing the “gdsl esterase/lipase” (1353594at33090) at the Viridiplantae level.

This example illustrates the cross-referencing capabilities of the tool, allowing researchers to compare annotations derived from the methods implemented in Hayai-Annotation. To reinforce the accuracy of these predictions, we conducted a manual search of the “nr” database at the Viridiplantae taxonomic level. The NCBI BLAST results (executed on November 11, 2024; see Supplementary File S2, Table 1) confirmed the predicted GDSL esterase/lipase function. While this is a single example, it highlights the core principle of Hayai-Annotation: the integration of multiple evidence streams and intuitive visualization to facilitate functional characterization across diverse plant species.

#### 3.5.2 Analysis of uncharacterized orthologous groups

**Fig. 4:**
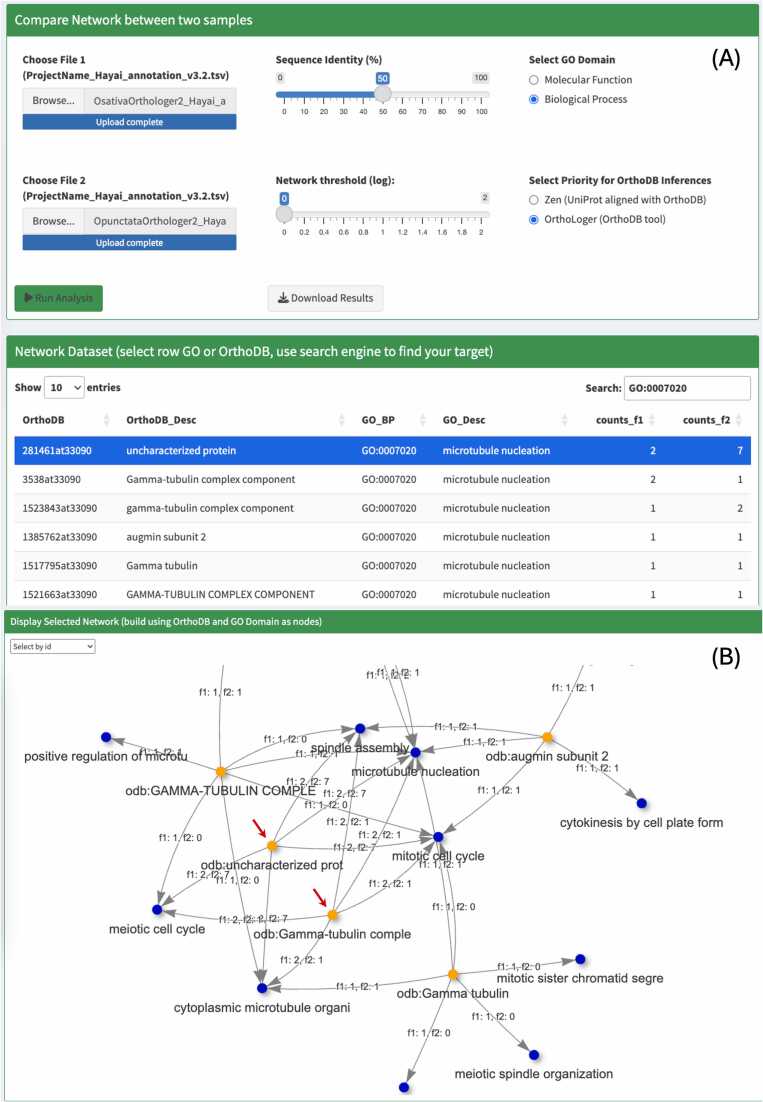
Network analysis module showing: (A) Input files and parameters, with sequence identity 50 %, GO domain set to “Biological Process”, and OrthoDB priority set to “OrthoLoger”. The search engine in the Network Dataset was used to select all the OGs with GO 0007020. By selecting a line in the Network Dataset, a network is generated using the GO term as query to find the associated OGs; and (B) the network generated with the selected parameters, with the OGs “odb:uncharacterized prot” and “odb:Gamma-tubulin complex” shown in orange, and the shared GO (BP) terms associated with these orthologs shown in blue. Note: “odb” is an abbreviation for OrthoDB.

The Network Analysis module facilitated the exploration of uncharacterized OGs associated with specific biological processes or molecular functions. For example, when analyzing the biological process “microtubule nucleation” (GO:0007020) with parameters set to Biological Process (BP) and OrthoLoger priority, the network revealed two OGs associated with this GO term: “uncharacterized protein” (281461at33090) and “Gamma-tubulin complex component” (3538at33090) (Fig. 4).

Due to truncation for visualization purposes in the network display, the full OG names and IDs were retrieved from the Network Dataset. The counts_f1 and counts_f2 columns represent the total number of genes annotated with the corresponding OG–GO (BP) for *O. sativa* and *O. punctata*, respectively. The network generated with the selected parameters shows the OGs “uncharacterized prot” and “Gamma-tubulin complex”, and the GO (BP) terms associated with these orthologs, suggesting that these OGs may share similar biological process.

**Fig. 5:**
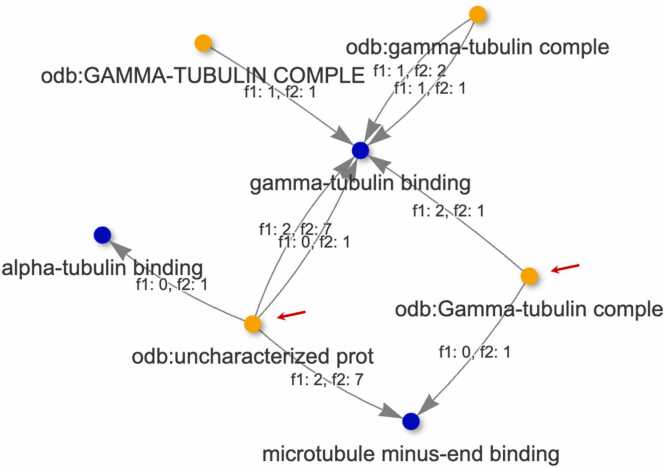
The network generated with the GO domain molecular function and prioritizing OrthoLoger for OG inferences. The OGs “odb:uncharacterized prot” (281461at33090) and “odb:Gamma-tubulin complex” (3538at33090) are indicated by a red arrow. The shared GO (MF) terms associated with these orthologs, “gamma-tubulin binding” (GO:0043015) and “microtubule minus-end binding” are shown in blue (GO:0051011).

To identify the genes in each species, a search was conducted in the “Main Annotation File 1” and “Main Annotation File 2” at the bottom of the webpage, with OG 281461at33090. This search revealed the product name “Gamma-tubulin complex component” along with the same molecular function GO IDs: GO:0043015 (gamma-tubulin binding) and GO:0051011 (microtubule minus-end binding). These annotations are also displayed in the network generated based on the molecular function GO domain (Fig. 5), providing strong support for the effectiveness of OG-GO visualization in enhancing the characterization of orthologs.

This example demonstrates how the Network Analysis module in Hayai-Annotation enables researchers to quickly identify and compare orthologous groups and their associated functions, through a single, user-friendly interface. By unifying data in one environment, the tool streamlines filtering (such as, sequence identity, selecting a GO domain—BP or MF—for network reconstruction, or considering differences in gene counts between two species), constructing OG–GO networks, and displaying the corresponding filtered data in a tabular format. This integrated approach allows scientists to efficiently generate functional hypotheses for previously uncharacterized orthologs. These features are expected to be particularly valuable for database curators tasked with comparing annotations across different species. Although these inferences represent initial steps based on limited conditions, they can still spark more detailed investigations, ultimately fostering a richer understanding of gene functions among plants.

#### 3.5.3 Analysis of gene ontology (GO) propagation

**Fig. 6:**
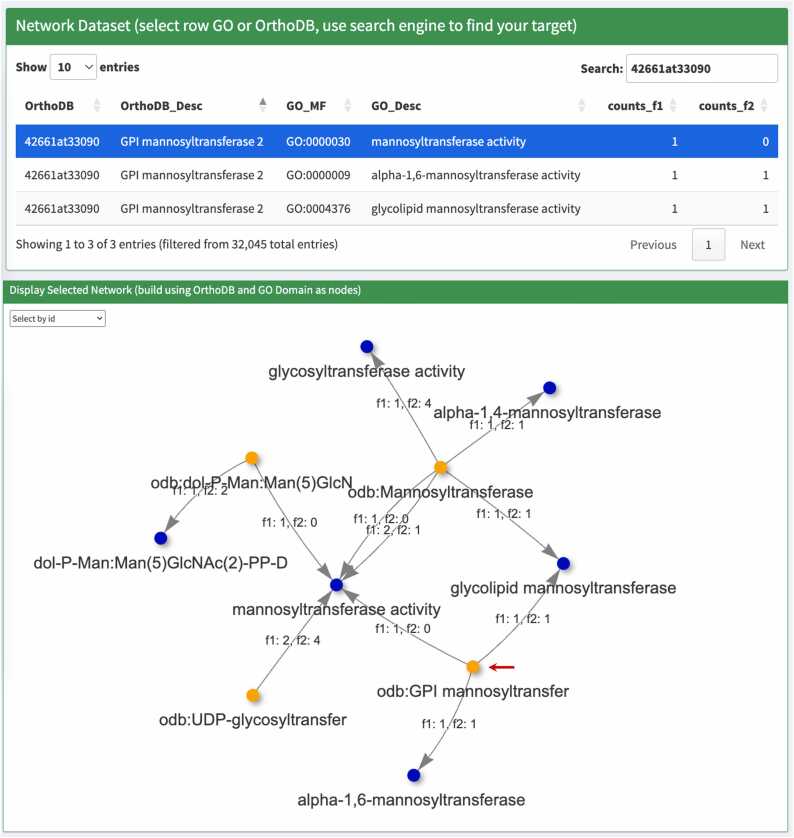
The network generated using the GO domain molecular function, with OrthoLoger prioritized for OG inferences. The OGs “odb:GPI mannosyltransferase 2” (42661at33090) are indicated by a red arrow. The GO-MF terms associated with this ortholog are shown in blue: mannosyltransferase activity, alpha-1,6-mannosyltransferase activity and glycolipid mannosyltransferase activity. The counts_f1 and counts_f2 correspond to the number of proteins annotated in *O. sativa* and *O. punctata*, respectively.

We next investigated the potential propagation of Gene Ontology (GO) annotations among closely related proteins in *O. sativa* and *O. punctata* using the Network Analysis module. The parameters selected comprised the molecular function domain with OrthoDB annotations prioritized via OrthoLoger. The GO ID GO:0000030 (mannosyltransferase activity) was queried in the network dataset, and a row from the displayed table was selected to display the corresponding network (Fig. 6).

Within the network, the ortholog GPI mannosyltransferase 2 (42661at33090) (red arrow) stood out. By examining the network edges (f1, f2) or the dataset (counts_f1 and counts_f2) for *O. sativa* and *O. punctata*, respectively, we investigated the number of proteins associated with the OG 42661at33090 and its GO annotations. In *O. sativa* three distinct GO terms were identified, alpha-1,6-mannosyltransferase activity (GO:0000009), glycolipid mannosyltransferase activity (GO:0004376), and mannosyltransferase activity (GO:0000030). However, *O. punctata* lacked the annotation for mannosyltransferase activity.

Searching for the OG 42661at33090 on the main annotation files at the bottom of the browser reveals the corresponding proteins for each species. In *O. sativa*, the protein Os12t0498700–01, UniProt accession Q2QQC9, and *O. punctata*, OPUNC12G11540.1 identified as A0A0E0MMN5, were found to precisely match the annotated proteins at the UniProtKB website. Upon closer examination of the UniProt entries, accessed by clicking the accessions hyperlinks in the Hayai-Annotation tables, we noticed that the first two GO annotations were electronically inferred from InterPro signatures (IEA:InterPro), while the third annotation (GO:0000030) was inferred from biological ancestry (IBA:GO_Central). Given that these proteins belong to the same genus, it is plausible that OPUNC12G11540.1 may also inherit the molecular function mannosyltransferase activity (GO:0000030).

#### 3.5.4 Case study of gene loss and gain

We examined NusB domain-containing proteins, which have been implicated in transcriptional regulation and potential horizontal gene transfer (HGT) events from plant-associated bacteria [29]. In the Hayai-Annotation Network Analysis module, after prioritizing OrthoLoger for OrthoDB inferences, we searched for the Pfam ID PF01029 (NusB) in the Main Annotation File 1 and Main Annotation File 2, respectively, in *O. sativa* and *O. punctata*. Two OGs were identified, 45459at33090 (uncharacterized protein, column ODB_Description) and 1196620at33090 (SAM-dependent methyltransferase RsmB-F/NOP2-type, catalytic core), with the same Pfam domains PF01189 (Methyltr_RsmB-F), PF01029 (NusB), and PF22458 (RsmF-B_ferredox). Cultivated rice showed one protein Os09t0477900–01 (UniProt accession Q0J0Y4) with OG 45459at33090 and wild rice presented seven alternative splicings with two different proteins, OPUNC09G12440.1 (A0A0E0M2H0) and OPUNC09G12440.2 (A0A0E0M2H1), respectively 45459at33090 and 1196620at33090.

We compared our findings with the NusB domain-containing protein in *A. thaliana*, accession AT3G13180 (UniProt accession Q8VYC4), described in Haimlich et al. (2024), first using UniProt BLAST (https://www.uniprot.org/blast) against *O. sativa* and *O. punctata*, and then by performing a multiple alignment (https://www.uniprot.org/align). All alignment results and FASTA sequences are documented in Supplementary File S1 (Fig. 2, Fig. 3) and Supplementary File S2 (Table 2, Table 3, Table 4). The BLAST output showed the highest scores with Q0J0Y4 (*O. sativa),* A0A0E0M2H0 (*O. punctata*) and A0A0E0M2H1 (*O. punctata*): 64.2 %, 62.1 % and 54.0 % identity, respectively. The percent identity matrix, in the multiple alignment, showed that Q0J0Y4 has 97.63 % identity with A0A0E0M2H0, versus 89.13 % with A0A0E0M2H1. In addition, A0A0E0M2H0 showed 91.68 % identity with A0A0E0M2H1. These findings suggested that A0A0E0M2H1 might be an ancient duplication of A0A0E0M2H0. A search at the OrthoDB website (orthodb.org) displays the evolutionary profile for the OG 1196620at33090, which includes 22 genes in 16 species, indicating that *O. sativa* might have lost this gene.

## 4 Conclusion

Hayai-Annotation represents a significant advancement in the field of plant genome analysis, offering an innovative approach to functional gene annotation. By integrating DIAMOND sequence alignment and OrthoLoger ortholog inference, this tool achieves enhanced coverage in annotating genes. Hayai-Annotation is further distinguished by the inclusion of a Network Analysis module, which enables the exploration of conserved and species–specific biological processes through ortholog–GO term relationships. The development of Zen mapping notably expands the coverage of orthologous groups within UniProtKB-Plants, bridging gaps left by traditional methods and providing complementary insights alongside OrthoLoger. This dual approach ensures robust annotation coverage across diverse plant genomes, as demonstrated by case studies involving *A. thaliana, O. sativa, and O. punctata*. The tool’s capacity to identify orthologs even in species with less comprehensive database representation highlights its versatility and potential in addressing complex genomic challenges. By addressing key challenges in ortholog detection and GO annotation analysis, Hayai-Annotation will pave the way for deeper insights into plant genome evolution and functional genomics, ultimately accelerating research in plant biology and beyond.

## CRediT authorship contribution statement

**Sachiko Isobe:** Writing – review & editing. **Andrea Ghelfi:** Writing – original draft, Validation, Methodology, Formal analysis, Conceptualization.

## Declaration of Competing Interest

The authors declare no conflict of interest.
