# Supplementary material for: Hayai-Annotation: A functional gene prediction tool that integrates orthologs and gene ontology for network analysis in plant species
Source: Comput Struct Biotechnol J. 2024 Dec 16;27:117–26. doi: 10.1016/j.csbj.2024.12.011 (PMC11742577; doi:10.1016/j.csbj.2024.12.011)
Supplement: Supplementary file 1 — Supplementary material [file mmc1.docx]

**Supplementary Data.** **Hayai-Annotation: A functional gene prediction tool that integrates orthologs and gene ontology for network analysis in plant species**

**Authors**

Andrea Ghelfi^1*^, Sachiko Isobe^2^

1: National Institute of Genetics, Yata, 1111, Mishima, Shizuoka, 411-8540, Japan

2: Kazusa DNA Research Institute, Kazusa-Kamatari, 2-6-7, Kisarazu, Chiba, 292-0818, Japan (former). Graduate School of Agricultural and Life Sciences, The University of Tokyo, 1-1-1, Yayoi, Bunkyo, Tokyo, 113-8657, Japan

Corresponding Author

Andrea Ghelfi, E-mail: andreaghelfi@nig.ac.jp

National Institute of Genetics, Yata, 1111, Mishima, Shizuoka, 411-8540, Japan.

Tel.: +81-55-981-6853. Fax.: +81-55-981-6849

* 'To whom correspondence should be addressed'

**Evaluation of Zen OrthoDB Mapping: Benchmarking UniProtKB**

The UniProt-Plants dataset, downloaded on April 29, 2024, was analyzed to determine the percentage of entries in the TrEMBL and Swiss-Prot accessions that contain OrthoDB (v.11) annotations. This dataset was used as the ground truth for comparison with Zen mapping.

The script ‘extract_orthodb.py’ was used to extract UniProtKB accession IDs and their corresponding OrthoDB annotations from the UniProt data files (uniprot_sprot_plants.dat and uniprot_trembl_plants.dat). It generates two files: sp_acc2orthodb.txt (for Swiss-Prot) and tr_acc2orthodb.txt (for TrEMBL).

The comparison between UniProt-OrthoDB and Zen mapping was conducted using the Jupyter notebook script available in ‘evaluate_orthoDBv11.pdf.

Both scripts and the resulted Zen mapping table are publicly available on GitHub at ‘https://github.com/aghelfi/HayaiAnnotation/tree/main/evaluation’.

**Evaluation of Zen OrthoDB Mapping: Semantic Similarity Score**


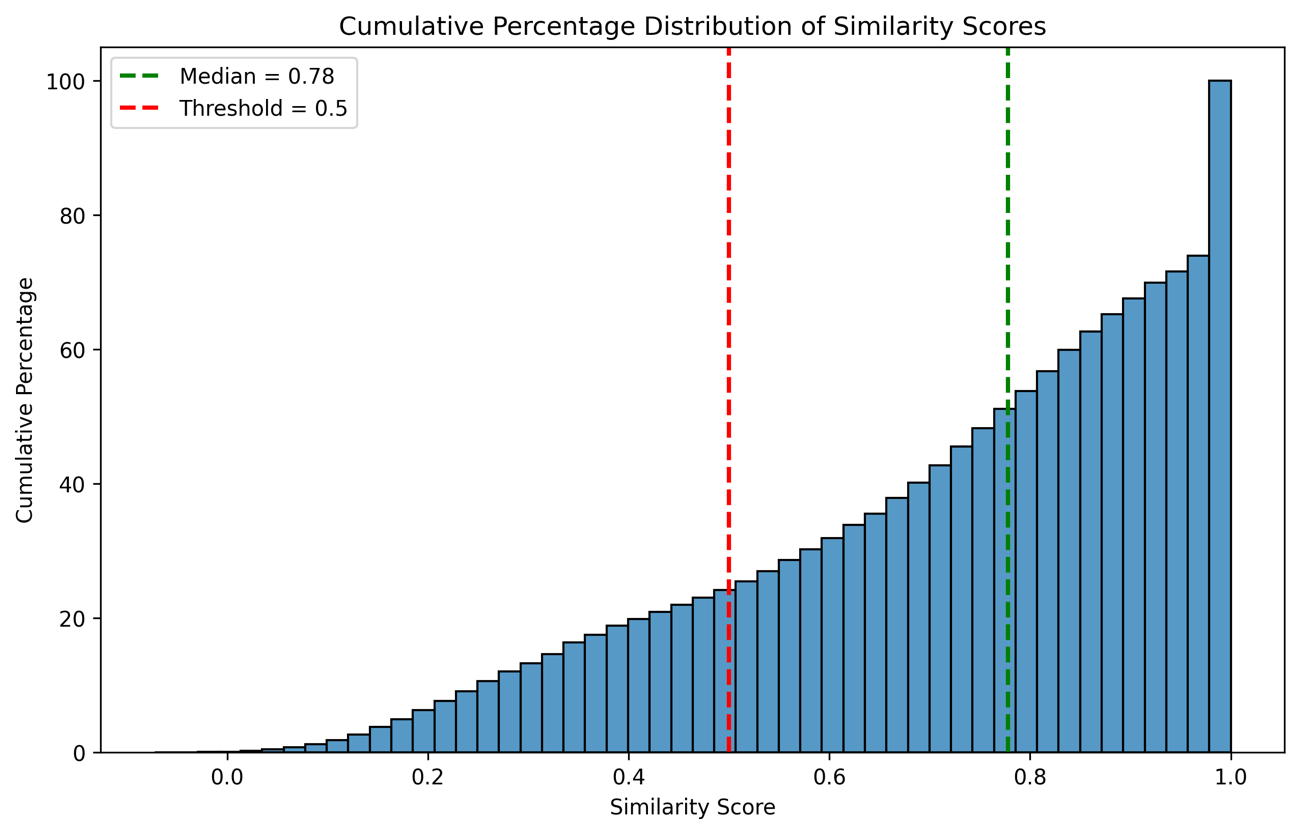


Supplementary File Figure 1. Distribution of semantic similarity scores between annotated protein product names in UniProtKB and their corresponding OrthoDB descriptions inferred using Zen mapping for *A. thaliana*. Similarity scores were computed using the SentenceTransformer model “pritamdeka/BioBERT-mnli-snli-scinli-scitail-mednli-stsb” on the output from Hayai-Annotation, focusing on the “Product_Name” and “Zen_OrthoDB_Desc” columns. The script calculate_prodName_similarity.pdf is available on GitHub at ‘https://github.com/aghelfi/HayaiAnnotation/blob/main/evaluation/’.

**Hayai-Annotation: Network Analysis Module**

**Case Study of Gene Loss and Gain: NusB domain-containing protein**

The study of the NusB domain-containing protein was conducted to validate the results of Hayai-Annotation. The analysis involved two main steps: a BLAST search and multiple sequence alignment, using UniProt BLAST (https://www.uniprot.org/blast/) and Align (https://www.uniprot.org/align/) webpages, executed on November 12, 2024. The results are displayed on:

**UniProt BLAST search**

Supplementary File S1 (this file), Figure 2: graphical representation.

Supplementary File S2, Table 2: BLAST result in TSV format.

**UniProt Align: Multiple sequence alignment**

Supplementary File S1 (this file), Figure 3. Multiple sequence alignment, in Clustal format, from the multiple sequence alignment using UniProt Align (default parameters, https://www.uniprot.org/align) with NusB proteins from *A. thaliana* (AT3G13180), *O. sativa* (Q0J0Y4), and *O. punctata* (A0A0E0M2H0 and A0A0E0M2H1).

Supplementary File S2, Table 3. NusB proteins from *A. thaliana* (AT3G13180), *O. sativa* (Q0J0Y4), and *O. punctata* (A0A0E0M2H0 and A0A0E0M2H1), in FASTA format.

Supplementary File S2, Table 4. Percent Identity Matrix for NusB proteins from *A. thaliana*, *O. sativa*, and *O. punctata*. Generated by Clustal 2.1 on the UniProt Align webpage.


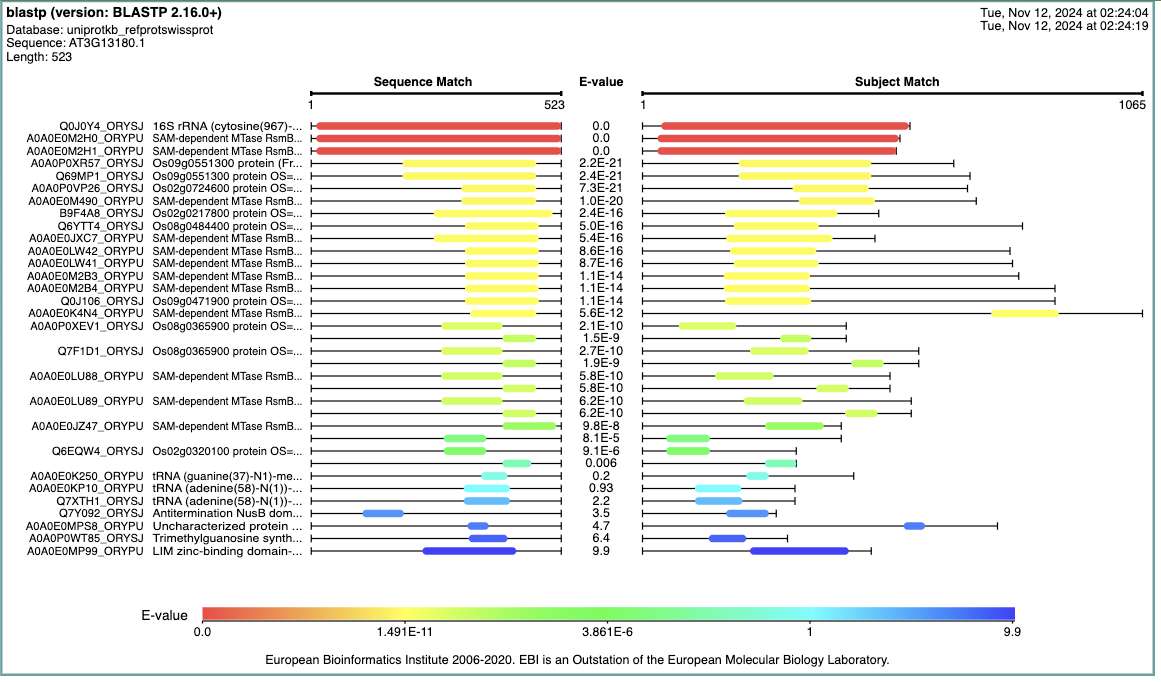


Supplementary File S1 Figure 2. Graphical representation from the manual annotation using UniProt BLAST with *A. thaliana* protein AT3G13180 (UniProt accession Q8VYC4), as described in Haimlich et al. (2024), against *O. sativa* and *O. punctata* (executed on November 12, 2024). Downloaded using the visual SVG format, (https://www.uniprot.org/blast/).

.

Supplementary File S1 Figure 3. Multiple sequence alignment, in Clustal format, from the multiple sequence alignment using UniProt Align (default parameters, https://www.uniprot.org/align) with NusB proteins from *A. thaliana* (AT3G13180), *O. sativa* (Q0J0Y4), and *O. punctata* (A0A0E0M2H0 and A0A0E0M2H1) (executed on November 12, 2024).
